# Role of the interferons in CD64 and CD169 expressions in whole blood: Relevance in the balance between viral‐ or bacterial‐oriented immune responses

**DOI:** 10.1002/iid3.289

**Published:** 2020-02-07

**Authors:** Pénélope Bourgoin, Géraldine Biéchelé, Inès Ait Belkacem, Pierre‐Emmanuel Morange, Fabrice Malergue

**Affiliations:** ^1^ Department of Research and Development Immunotech‐Beckman Coulter Marseille France; ^2^ C2VN INSERM‐INRAE Aix‐Marseille University Marseille France; ^3^ UMR 7280, Center for Marseille‐Luminy Immunology (CIML) Marseille France; ^4^ Laboratory of Hematology La Timone Hospital (AP‐HM) Marseille France

**Keywords:** CD169, CD64, flow cytometry, interferons, pathways

## Abstract

**Introduction:**

CD64 expression increases on neutrophils during bacterial infections. Recently an increase in CD169 expression has been discovered on monocytes during viral infections. Generally, interferons α (IFNsα) and IFNsγ are key drivers of the infectious host immune response. The purpose of this study was to explore if a link exists between these IFNs and both biomarkers.

**Methods:**

Whole blood samples from healthy volunteers were stimulated with either IFNs, interleukins, or infectious extracts, to mimic an infectious state. Expressions of CD64 and CD169 were assessed in these samples by multiple flow cytometry methods, over precise kinetics.

**Results:**

The expression of CD64 was statistically higher in samples stimulated with IFNγ, and CD169 in those stimulated with IFNα (and all other type I IFNs). Surface expressions are directly induced by their respective IFNs via Janus kinase/signal transducer and activator of transduction pathways within 6 to 8 hours of incubation. Mixing both types of IFNs seemed to indicate that they partially inhibit each other.

**Conclusions:**

The induction of CD169 on monocytes and CD164 on neutrophils by type I and type II IFNs confirms the relevance of these markers for assessing between a viral‐ vs bacterial‐oriented immune response.

## Graphical abstract

CD64 and CD169 are expressed in samples stimulated with interferon γ (IFNγ) and IFNα, indicating relevance for discriminating infections.

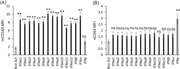

## 1 INTRODUCTION

To survive in its living environment, the human body is protected from infectious agents, their toxins, and the damage they can cause, by a set of effector cells and molecules. The innate immune system is an early but less‐specific biological mechanism that avoids the development of pathogenic organisms.^1^ Myeloid cells, such as monocytes and macrophages, neutrophils, or dendritic cells (DCs), have pattern recognition receptors (PRR) to immediately identify the pathogen‐associated or damage‐associated molecular patterns (PAMP or DAMP) from microorganisms. The binding of PAMP or DAMP to the PRR triggers a robust inflammatory response that leads to the activation of different signaling cascades depending on the nature of the infectious agent. Thus, among other things, it activates the production of antibacterial or antiviral mediators, such as interferon (IFN), as well as the expression of various molecules on the surface of the immune cells.^2^ Particularly, the secretion of type I IFNs (IFN I) has been suggested as a specific cellular response of viral infections and includes the IFN‐α family plus IFN‐β, IFN‐ω, IFN‐κ, and IFN‐ε. By contrast, only the unique type II IFN (IFN II), the IFN‐γ, mainly contributes to the clearance of bacterial infections.^3^

If IFN I and IFN II are clear signatures associated with viral and bacterial infections, their short life in the body fluids does not allow a robust differentiation.^4^ Thus, it appears that it would be useful to study molecules that are more stably expressed downstream of IFN activation.

CD64, also known as the high‐affinity immunoglobulin fragment crystallizable‐γ receptor 1, is mainly distributed on the surface of cells of myeloid lineage such as macrophages, monocytes, and DCs, for providing the first line of recognition and defense against invading microorganisms.^5^ It has almost no expression on neutrophils under healthy homeostasis. Upon bacterial infection, the activation of the immune system releases proinflammatory cytokines, including IFNγ, that highly induce the expression of CD64 on neutrophils (nCD64).^6^, ^7^, ^8^

Interestingly, recent works have identified sialoadhesin CD169 (Sn or SIGLEC‐1), a member of the sialic acid‐binding immunoglobulin‐like lectins (SIGLEC) family, as having a role during viral infections.^9^, ^10^ CD169 has been shown to be expressed on the surface of DCs and monocytes after antiviral molecule release, and, more particularly, a twofold elevated upregulation of CD169 on monocytes (mCD169) was observed in vitro when induced by IFNα.^11^, ^12^

Thus, the combinatorial detection of CD64 and CD169, respectively, on the surface of neutrophils and monocytes, could be a specific measure for the distinction between the different causes of infections.^13^ The main goal of the study was to understand the link that exists between both biomarkers and IFNs produced in response to infections. The main hypothesis was that the understanding of this functional link could help demonstrate the relevance of using these biomarkers for discriminating between bacterial and viral infections.

## 2 MATERIALS AND METHODS

### 2.1 Study samples and activation testing

Immunotech evaluations were conducted on leftover heparin blood samples from healthy volunteers, obtained from the Saint Joseph Hospital (Marseille, France) and tested after informed consent and hospital Ethical Committee approval.

Samples were activated either with type I IFNs (IFNα1‐alpha A (2a), IFNα2‐alpha B2 (8), IFNα3‐alpha C (10), IFNα4‐alpha D (1 [Val(114)]), IFNα5‐alpha F (21), IFNα6‐alpha G (5), IFNα7‐alpha H2 (14), IFNα8‐alpha I (17), IFNα9‐alpha J1 (7), IFNα10‐alpha K (6), IFNα11‐alpha 4b (4), IFNα12‐alpha WA (16), IFNβ, and IFNω), used at 3 ng/mL, or with IFNγ, used at 30 ng/mL, all from R&D Systems (Minneapolis, MN).

For comparison, whole blood was also activated with interleukins (IL) (IL‐2, IL‐6, IL‐12, and IL‐18), used at 1000 U/mL, from R&D Systems, or infectious extracts, such as polyinosinic‐polycytidylic acid (Poly IC) or lipopolysaccharide (LPS), from *Escherichia coli* O127:B8, both at 10 µg/mL, from Sigma‐Aldrich Co, (St. Louis, MO).

All activations were made in closed tubes in a water bath at 37°C. When needed, samples were also coincubated with Brefeldin A (Bref. A), at 10 µg/mL, from Sigma‐Aldrich Co.

For in vivo comparison of these in vitro activations, leftover ethylenediaminetetraacetic acid blood samples from viral‐ and bacterial‐infected subjects, admitted to the Adult Emergency Unit at La Timone Hospital (Marseille, France), were tested within 4 hours of admission. Samples were obtained under informed consent and Ethical Committee approval (Committee for Protection of Persons n°181160; ID‐RCB n° 2018 A02706‐49).

### 2.2 Flow cytometry procedures

Samples were treated at room temperature with flow cytometry extracellular and/or intracellular and/or phospho‐epitopes procedures, with reagents that were for research use only and all used at their recommended doses. For each experiment, multiple individual samples were processed (number of tested donors indicated by n), with all testing in singlicate due to the known strong reproducibility of the procedures used.

The extracellular procedure has been described by Bourgoin et al^14^ in 2019. Briefly, 5 µL of whole blood is simultaneously lysed and stained at room temperature by incubating for 15 minutes in the dark, with 500 µL of the Versalyse lysing solution and the CD64‐CD169/infections dried custom mixture, composed of anti‐CD169‐phycoerythrin (PE) (clone 7‐239) and anti‐CD64‐Pacific Blue (PBE) (clone 22). The custom mixture was replaced either with anti‐IgG1‐PE and anti‐IgG1‐PBE, at the same concentrations, for staining controls, or with Annexin V‐fluorescein isothiocyanate and propidium iodide for cell death evaluation. All products or custom products come from Beckman Coulter Inc, (Brea, CA).

The intracellular procedure is the method described within the PerFix nc kit (Beckman Coulter Inc). Briefly, 50 µL of whole blood is first fixed with the kit reagent number 1 “R1” and incubated for 15 minutes, then simultaneously lysed, permeabilized, and stained by incubating for 15 minutes in the dark with the kit reagent number 2 “R2” and the CD64‐CD169/infections dried custom mixture. Finally, the sample is washed once with the kit reagent number 3 “R3.”

The phospho‐epitopes procedure is the method described within the PerFix EXPOSE kit (Beckman Coulter Inc). Briefly, 100 µL of whole blood is first fixed with the kit reagent number 1 “R1” by incubating for 10 minutes and is simultaneously permeabilized and lysed with the kit reagent number 2 “R2” by incubating for 10 minutes. After a wash step, sample is stained by incubating for 15 minutes in the dark with the kit reagent number 3 “R3” and the DCs dried custom mixture, composed of anti‐CD123‐PE cyanin 7 (clone SSDCLY107D2), anti‐CD3‐allophycocyanin (APC) (clone UCHT1), anti‐CD14‐APC (clone RMO52), anti‐CD19‐APC (clone J3‐119), anti‐CD56‐APC (clone N901‐NKH1), anti‐CD11c‐APC Alexa Fluor 700 (clone BU15), anti‐human leukocyte antigen‐DR (HLA‐DR)‐PBE (clone Immu 357) and anti‐CD45‐Krome Orange (clone J33), all products from Beckman Coulter Inc. When needed, anti‐phospho‐epitope antibodies were also added: anti‐phosphorylated signal transducer and activator of transcription (pSTAT) 1‐Alexa Fluor 488 (clone 58D6), or pSTAT2‐PE (clone D3P2P) from Cell Signaling Technology Inc, (Danvers, MA). Finally, the sample is washed once with the kit reagent number 4 “R4.”

### 2.3 Flow cytometry and statistical analyses

All data were collected on a 3‐laser, 10‐color Navios flow cytometer and analyzed using Kaluza Software version 2.1 (both from Beckman Coulter Inc).

When using CD64‐CD169/infections dried custom mixture, monocytes and neutrophils were first gated on the basis of their typical forward‐ and side‐scatter characteristics. CD169 and CD64 were respectively measured on the surface of the gated cells on monoparametric histograms.

When using the DCs dried custom mixture, leukocytes were first gated on their CD45 expression. Monocytes and neutrophils were gated out on the basis of their side‐scatter characteristics whereas DCs were isolated based on their additional HLA‐DR+ CD3− CD14− CD19− CD56− expression. Myeloid DCs (mDCs) were differentiated from plasmacytoid DCs (pDCs) on their respective CD11c+ or CD123+ expressions. Finally, phosphoepitope expressions were measured on monocytes, neutrophils, mDCs, or pDCs.

All results were given as a median of fluorescence intensity related to the entire population.

Comparisons of quantitative variables were performed using paired Tukey or Dunnett's control tests and by analysis of variance for more than two groups. The statistical analyses were performed using Jump software version 10 (SAS Institute Inc, Cary, NC). All *P* values are considered statistically significant under .05.

## 3 RESULTS

### 3.1 CD169 and CD64 expressions are induced by IFN stimuli

In the preamble to the study, natural in vivo expressions of CD169 at the surface of monocytes and of CD64 at the surface of neutrophils were assessed in healthy and viral‐ or bacterial‐infected subjects (Figure 1). In comparison to healthy subjects, results showed that mCD169 expression was increased from 0.60 (±0.07) to 7.44 (±3.39) in viral‐infected subjects (n = 5), and nCD64 expression was increased from 0.64 (±0.09) to 1.37 (±0.19) in bacterial‐infected subjects (n = 5), whereas no change was measured in the respective isotypic control tubes.

Figure 1CD169 and CD64 in vivo expressions in whole blood. Either IgG1‐PE and IgG1‐PBE (isotype; A1,A2), or CD169‐PE and CD64‐PBE (staining; B1) expressions were respectively measured on the surface of the monocytes and of the neutrophils in the whole blood of five healthy subjects, five viral‐infected subjects, and five bacterial‐infected subjects. Results are expressed as averages ± standard deviations of their median of fluorescence intensities (MFI). Examples were given for one first healthy volunteer whole blood, one‐second viral‐infected whole blood, and one‐third bacterial‐infected whole blood

**Figure:**
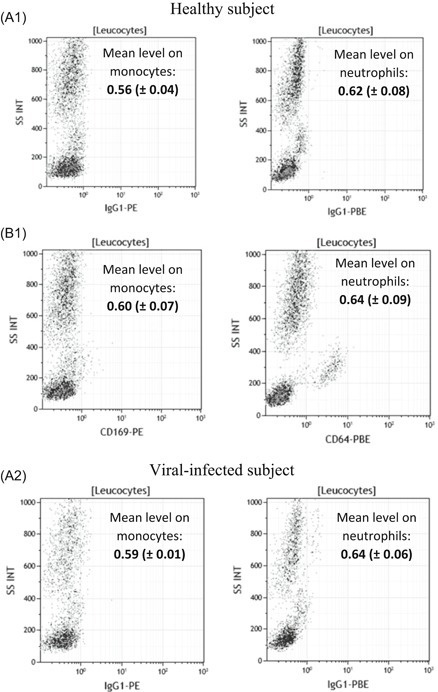

**Figure:**
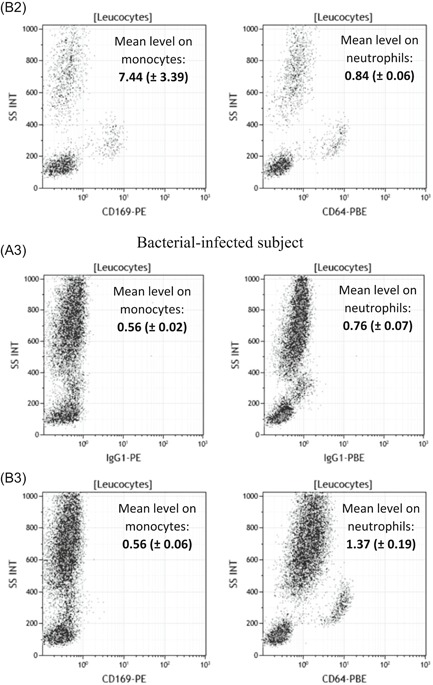

**Figure 2:**
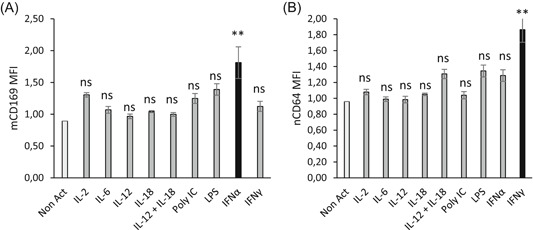
CD169 and CD64 expressions after blood stimulation. Whole blood of three donors was coincubated for 7 hours at 37°C with either no activator (Non Act), or interleukins (IL‐2, IL‐6, IL‐12, IL‐18, IL‐12 + IL‐18) or infectious extracts (Poly IC, lipopolysaccharide [LPS]) or interferons (IFNα1 and IFNγ). Extracellular staining of the activated blood was performed with the CD64‐CD169/infections antibody mixture. Results are expressed as averages ± standard deviations of the median of fluorescence intensities (MFI) of CD169 on monocytes (mCD169) (A) and of CD64 on neutrophils (nCD64) (B). The comparison was made using a Dunnett's control test, with the Non‐Act condition used as control (in white) and *P* value was considered either not statistically significant above .05 (in gray; NS) or statistically significant under .05 (in black; *) or under .01 (in black; **). NS, not significant

The first part of the study was to quantify and statistically compare these same expressions when whole blood is coincubated in vitro with several activators. Since most of them are known to induce multiple responses and the release of other soluble mediators, a short incubation time was chosen to limit the risk of measuring indirect effects. At 3 and 5 hours, no specific effects were detected (except LPS nonspecific early response), whereas the first significant changes were observed at 7 hours (Figure 2). All detailed values are given in Table S1.

After 7 hours of incubation, mCD169 expression was significantly increased in whole blood activated by IFNα1 (1.8 ± 0.6, *P* = .006; n = 3), in comparison to a nonactivated whole blood (0.9 ± 0.1), whereas it was unchanged when incubated with other activators (IL‐2 = 1.3 ± 0.1, *P* = .2011; IL‐6 = 1.1 ± 0.2, *P* = .9219; IL‐12 = 1.0 ± 0.1, *P* = .9999; IL‐18: 1.0 ± 0.1, *P* = .9674; IL‐12 + IL‐18 = 1.0 ± 0.1, *P* = .9964; Poly IC = 1.3 ± 0.1, *P* = .3324; LPS = 1.4 ± 0.3, *P* = .0857; IFNγ = 1.1 ± 0.1, *P* = .7634).

Conversely, nCD64 expression was significantly increased in whole blood activated by IFNγ (1.9 ± 0.4, *P* < .0001; n = 3), in comparison to a nonactivated whole blood (1.0 ± 0.1), whereas it was unchanged when incubated with other activators (IL‐2 = 1.1 ± 0.1, *P* = .9515; IL‐6 = 1.0 ± 0.1, *P* = 1.0000; IL‐12 = 1.0 ± 0.1, *P* = 1.0000; IL‐18 = 1.1 ± 0.1, *P* = .9910; IL‐12 + IL‐18 = 1.3 ± 0.2, *P* = .1228; Poly IC = 1.0 ± 0.1, *P* = .9962; LPS = 1.3 ± 0.1, *P* = .0725; IFNα1 = 1.3 ± 0.2, *P* = .1597).

After 24 hours of incubation, some activators induced increased responses, indicating they had later effects on the blood samples, probably via indirect pathways. Therefore, the results of this screening indicated that IFNs have earlier, more direct and more persistent potential implications in biomarker expression.

**Figure 3:**
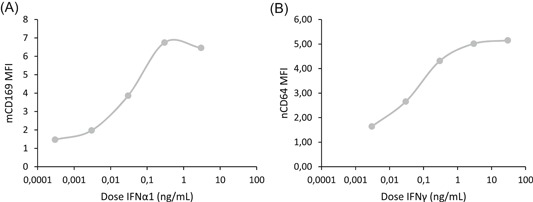
Dose‐response curves of interferon α1 (IFNα1) and IFNγ. The whole blood of two donors was coincubated for 15 hours at 37°C with five doses (between 0.0003, 0.003, 0.03, 0.3, 3, or 30 ng/mL) of either one type I IFN (IFNα1) or one type II IFN (IFNγ). Extracellular staining of the activated blood was performed with the CD64‐CD169/infections antibody mixture. Results were expressed as averages of the median of fluorescence intensities (MFI) of CD169 on monocytes (mCD169) (Figure 2A) and of CD64 on neutrophils (nCD64) (Figure 2B)

Dose‐response curves for IFNα1 and IFNγ were then established to determine the optimum dose to maximize mCD169 and nCD64 expression, respectively (Figure 3). Longer incubation time was tested to increase the observed effects (15 vs 7 hours). Results were referring to dose‐response curves: optimal IFNα1 dose was 3 ng/mL, whereas the optimal IFNγ dose was 30 ng/mL. Both doses were similar to what could be released physiologically in the human body after bacterial or viral infections.^15^

In addition, mCD169 and nCD64 expressions, following stimulation by these optimum IFN doses, were compared to biomarker natural expressions in healthy and viral‐ or bacterial‐infected subjects (Figure 4). The main result was that IFNα1 and IFNγ, at their optimal doses, induced expression of CD169 on monocytes and of CD64 on neutrophils, respectively, at levels similar to what happens physiologically during the course of natural viral or bacterial infections. CD64 on monocytes (mCD64) was also moderately increased upon IFNγ stimulation, as observed in bacterial‐infected subjects; however, mCD64 has been demonstrated in many studies to be less accurate than nCD64.^16^ This study thus focused only on nCD64.

Figure 4CD169 and CD64 flow cytometry expressions on natural or incubated blood. First, leukocytes were isolated from red blood cells or debris or apoptotic cells based on their typical side (SS INT) and forward scatter (FS INT). Then, CD169 and CD64 were respectively measured on the surface of the monocytes (CD169 on monocytes) and of the neutrophils (CD64 on neutrophils). Examples of their expressions were given for three subjects: A, One first healthy volunteer whole blood. B, The same healthy volunteer whole blood that was not activated by interferons (IFNs), but that staid 15 hours at 37°C. C, The same healthy volunteer whole blood that was activated by IFNα1 for 15 hours at 37°C. D, The same healthy volunteer whole blood that was activated by IFNγ for 15 hours at 37°C. E, One‐second viral‐infected whole blood, and (F) one‐third bacterial‐infected whole blood

**Figure:**
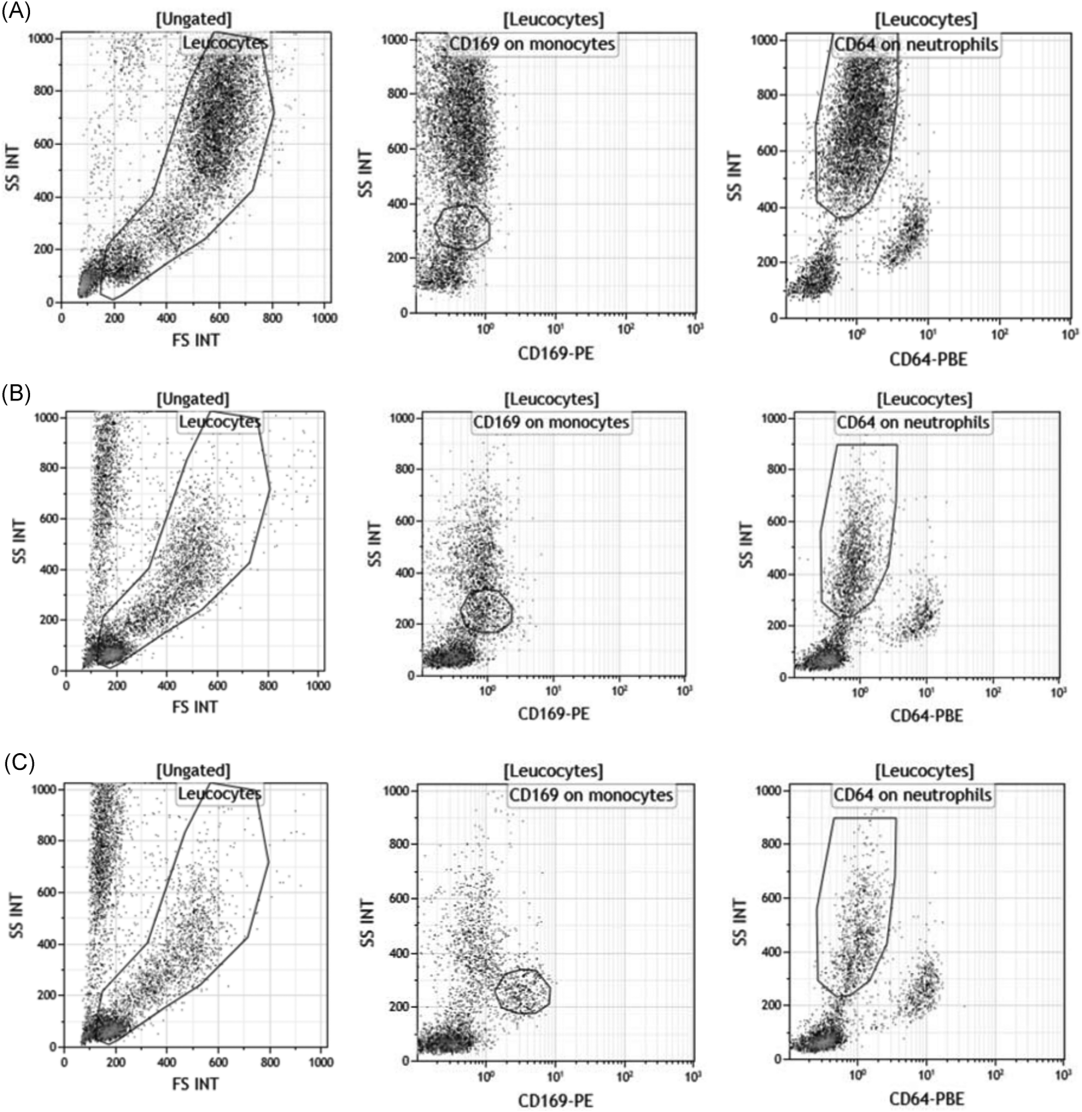

**Figure:**
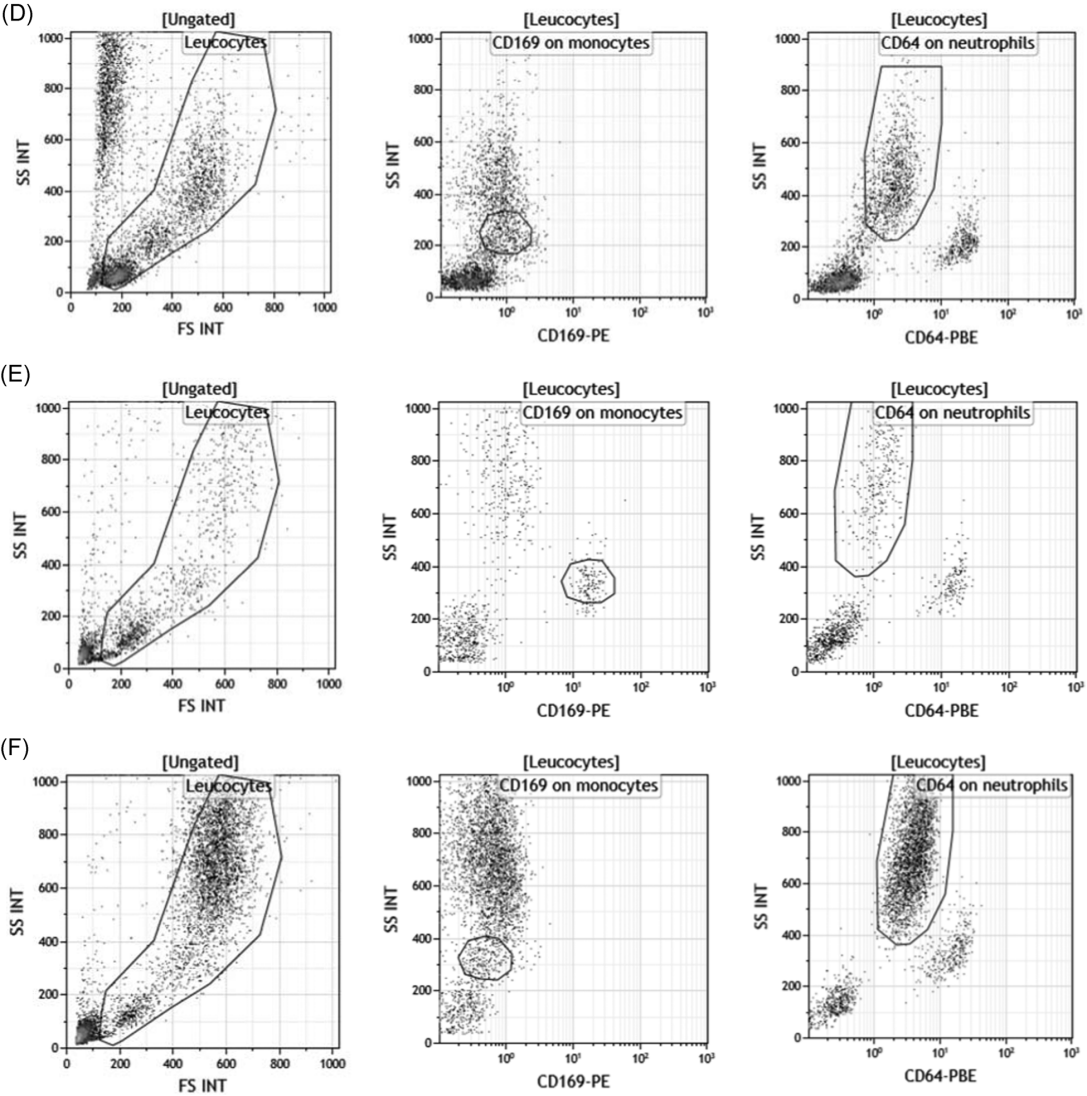

The plots also showed that keeping the whole blood of a healthy volunteer in a water bath at 37°C for 15 hours did not induce a nonspecific activation of either biomarker on any cell subsets, except a slight increase of mCD169.

Finally, this long ex vivo incubation induced some cell death that might interfere with the results. However, Figure S1 demonstrated that apoptotic or dead cells account for less than 5% of the total sample, and including them or not in the analysis did not change the biomarker levels.

### 3.2 CD169 expression is induced by IFN type I and CD64 expression is induced by IFN type II

**Figure 5:**
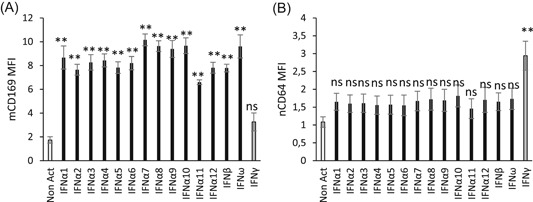
CD169 and CD64 expression after interferon (IFN) stimulation. Whole blood of four donors was coincubated for 15 hours at 37°C with either no IFN (Non Act), or type I IFNs (IFNα1, IFNα2, IFNα3, IFNα4, IFNα5, IFNα6, IFNα7, IFNα8, IFNα9, IFNα10, IFNα11, IFNα12, IFNβ, and IFNω) or type II IFN (IFNγ). Extracellular staining of the activated blood was performed with the CD64‐CD169/infections antibody mixture. Results are expressed as averages ± standard deviations of the median of fluorescence intensities (MFI) of CD169 on monocytes (mCD169) (Figure 4A) and of CD64 on neutrophils (nCD64) (Figure 4B). Comparison was made using a Dunnett's control test, with the Non Act condition used as control (in white), and *P* value was considered either not statistically significant above .05 (in gray; NS) or statistically significant under .05 (in black; *) or under .01 (in black; **). NS, not significant

Based on the previously determined doses, a larger spectrum of each IFN family was tested: IFNα1 belongs to the IFN I family which includes 13 subtypes of IFNα (only 1‐12 were tested), plus IFNβ and IFNω, whereas IFNγ is the unique IFN II (Figure 5). The longer incubation time was kept (15 vs 7 hours). Detailed values are given in Table S2.

Interestingly, mCD169 expression was again significantly increased in whole blood activated by IFNα1 (8.7 ± 1.9, *P* < .0001; n = 4), in comparison to a nonactivated whole blood (1.7 ± 0.5), but also by all IFNα subtypes (IFNα2 = 7.7 ± 0.9, *P* < .0001; IFNα3 = 8.3 ± 1.3, *P* < .0001; IFNα4 = 8.4 ± 1.1, *P* < .0001; IFNα5 = 7.8 ± 1.0, *P* < .0001; IFNα6 = 8.2 ± 1.1, *P* < .0001; IFNα7 =  10.2 ± 1.0, *P* < .0001; IFNα8 = 9.6 ± 0.9, *P* < .0001; IFNα9 = 9.4 ± 1.4; *P* < .0001; IFNα10 = 9.7 ± 1.3, *P* < .0001; IFNα11 = 6.6 ± 0.4, *P* = .0005; IFNα12 = 7.8 ± 0.9, *P* = .0042) and by others in the IFN I family (IFNβ = 7.8 ± 0.6, *P* < .0001; IFNω = 9.6 ± 1.9, *P* < .0001). mCD169 expression, however, remained low when incubated with IFN II (3.3 ± 1.5, *P* = .1979).

Conversely, nCD64 expression was again significantly increased in whole blood activated by IFNγ (2.9 ± 0.8, *P* = .0008; n = 4), in comparison to nonactivated whole blood (1.1 ± 0.3), but not when incubated with the IFN I family (IFNα1 = 1.7 ± 0.5, *P* = .8185; IFNα2 = 1.6 ± 0.5, *P* = .8897; IFNα3 = 1.6 ± 0.5, *P* = .8743; IFNα4 = 1.6 ± 0.5, *P* = .9318; IFNα5 = 1.6 ± 0.5, *P* = .9199; IFNα6 = 1.6 ± 0.6, *P* = .9384; IFNα7 = 1.7 ± 0.5, *P* = .7835; IFNα8 = 1.7 ± 0.6, *P* = .6995; IFNα9 = 1.7 ± 0.6, *P* = .7549; IFNα10 = 1.8 ± 0.6, *P* = .5375; IFNα11 = 1.5 ± 0.6, *P* = .9882; IFNα12 = 1.7 ± 0.7, *P* = 1.0000; IFNβ = 1.7 ± 0.5, *P* = .8147; IFNω = 1.7 ± 0.6, *P* = .6821).

These results indicated that all IFN I had a direct influence on the expression of CD169 on monocytes and that IFN II was involved in CD64 expression on neutrophils.

### 3.3 CD169 and CD64 biomarkers have rapid expression kinetics after IFN stimuli

**Figure 6:**
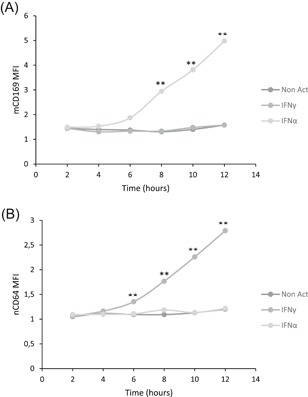
CD169 and CD64 kinetics after interferon (IFN) stimulation. Whole blood of four donors was coincubated for 2 to 12 hours at 37°C with either no IFN (Non Act; in the dark gray), or one type II IFN (IFNγ; in medium gray) or one type I IFN (IFNα1; in light gray). Extracellular staining of the activated blood was performed with the CD64‐CD169/infections antibody mixture. Results were expressed as averages of the median of fluorescence intensities (MFI) of CD169 on monocytes (mCD169) (Figure 5A) and of CD64 on neutrophils (nCD64) (Figure 5B). The comparison was made using an analysis of variance test, for which *P* value was considered statistically significant under .05. When *P* value was under .05, a comparison was made using a paired Tukey test, for which *P* value was also considered statistically significant under .05 (*) or under .01 (**)

In the second part of the study, precise kinetics of both biomarkers were studied. In preparation for the study, the hypothesis that the response amplitude might depend on the blood volume incubated was tested (Figure S2). After comparing several IFN‐activated blood volumes, results showed that these long‐term activations by IFN should be done in a whole blood volume of at least 300 µL, otherwise the mean measured level could be lowered. This could be explained by the fact that a smaller volume of blood, placed in 5 mL closed test tubes for incubation with IFNs, is unable to equilibrate CO_2_ and/or humidity with the larger volume of air (4 to 5 mL), which changes the cellular environment too much (pH and/or osmolality) for a proper activation of these markers. Having this new information, CD64 and CD169 biomarker kinetics were established by activating an amount of blood from 300 to 500 µL with IFN (Figure 6). Detailed values are given in Table S3.

As previously found, mCD169 was specifically induced on monocyte surfaces after IFN I stimulation (*P* < .0001) but not after IFN II (*P* = .1706) or no activation (*P* = .1299). When statistically comparing incubation time, results showed that expression of mCD169 was significantly increased by IFN I after 8 hours of incubation (8 hours: 2.9 ± 0.3; 10 hours: 3.8 ± 0.3; 12 hours: 4.98 ± 0.2; all *P* < .001; n = 4) in comparison to 2 hours (1.5 ± 0.1), 4 hours (1.5 ± 0.2), or 6 hours (1.9 ± 0.1).

In the same way, nCD64 was induced on the neutrophil surface after IFN II stimulation (*P* < .0001) but not after IFN I (*P* = .2456) or no activation (*P* = .3776). When statistically comparing incubation time, results showed that expression of nCD64 was significantly increased by IFN II after 6 hours of incubation (6 hours: 1.3 ± 0.1; 8 hours: 1.8 ± 0.1; 10 hours: 2.3 ± 0.1; 12 hours: 2.8 ± 0.1; all *P* < .001; n = 4) in comparison to 2 hours (1.1 ± 0.1) or 4 hours (1.2 ± 0.1).

Biomarker expression was observed as early as 6 hours, which did not exclude that their expression might be indirectly associated to IFN stimuli through various signaling cascades.

### 3.4 CD169 and CD64 biomarkers have direct expression kinetics after IFN stimuli

**Figure 7:**
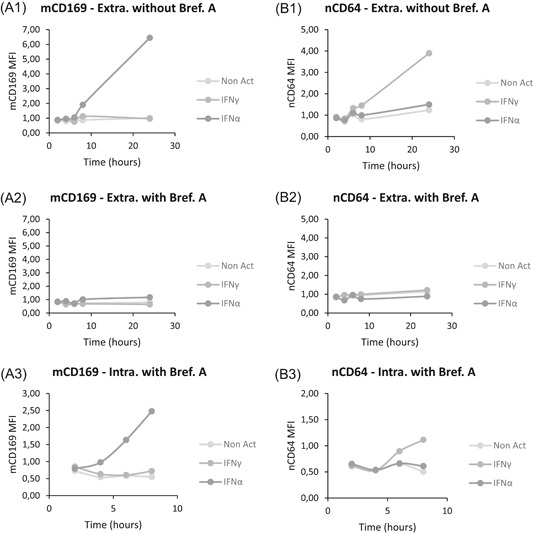
Direct or indirect CD169 and CD64 kinetics after interferon (IFN) stimulation. Whole blood of eight donors was coincubated for 2 to 24 hours at 37°C with either no IFN (Non Act; in light gray), or one type II IFN (IFNγ; in medium gray) or one type I IFN (IFNα1; in the dark gray). On the one hand, biomarker extracellular expressions (Extra.) were assessed in activated whole blood without Brefeldin A (Bref. A), a Golgi apparatus blocker, (A1 and B1) or with Brefeldin A (A2 and B2). On the other hand, intracellular expressions (Intra.) were evaluated with Brefeldin A only (A3 and B3). All stainings were performed with the CD64‐CD169/infections antibody mixture. Results were expressed as averages of the median of fluorescence intensities (MFI) of CD169 on monocytes (mCD169) (Figure 6A) and of CD64 on neutrophils (nCD64) (Figure 6B). The comparison was made using an analysis of variance test, for which *P* value is considered statistically significant under .05

**Figure 8:**
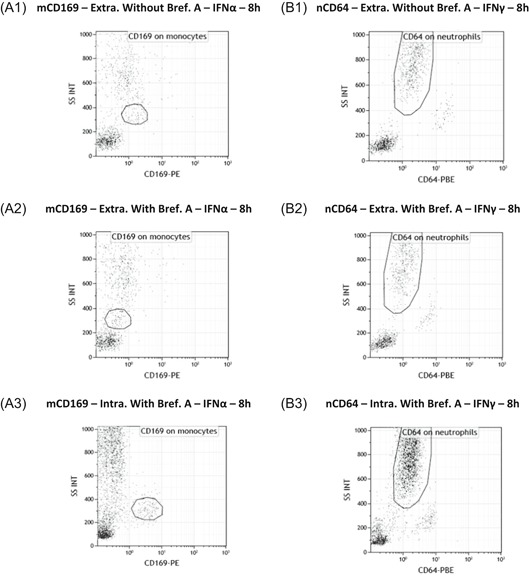
Flow cytometry extracellular or intracellular expressions of CD169 and CD64. Flow cytometry example of whole blood of one donor that was coincubated for 8 hours at 37°C with either one type I interferon (IFNα1) (Figure 7A) or one type II IFN (IFNγ) (Figure 7B). CD169 and CD64 were respectively presented on monocytes (mCD169) (Figure 7A) and neutrophils (nCD64) (Figure 7B). On the one hand, biomarker extracellular expressions (Extra.) were showed in activated whole blood without Brefeldin A (Bref. A) (A1 and B1) or with Brefeldin A (A2 and B2). On the other hand, intracellular expressions (Intra.) were showed with Brefeldin A only (A3 and B3)

It has been described that many cytokines are released within 2 to 4 hours poststimulation.^17^ The IFNs were thus evaluated to understand if they directly or indirectly activate the expression of CD64 and CD169 (Figure 7). Detailed values are given in Table S4, and results are supported by flow cytometry plots presented in Figure 8.

The hypothesis was tested by using Brefeldin A, a Golgi apparatus blocker that acts by blocking exocytosis of molecules, preventing both the secretion of soluble proteins (such as cytokines) and also the surface expression of de novo synthesized proteins (such as CD64 and CD169). If IFNs directly activate the cells to produce the biomarkers, then Brefeldin A would prevent their expression at the surface, and they would remain intracellular. However, if IFNs activate intermediary actors that produce intermediary molecules that would finally stimulate the cells to express the biomarkers, then Brefeldin A would cause intracellular retention of the first intermediary molecules, and there would be no activation of the final cell to either intracellularly produce or extracellularly express the biomarkers.

When quantifying the extracellular expression of mCD169 and nCD64 without Brefeldin A (Figure 7A1,B1), results showed significant increases after IFN I (*P* < .0001) or IFN II (*P* = .0002) stimulations, respectively. On flow cytometry plots (Figure 8A1,B1), CD169 and CD64 extracellular expressions were also increased.

If Brefeldin A was added (Figure 7A2,B2), blocking of surface expression occurred as expected, as no significant changes in the expressions were observed (mCD169; *P* = .3478; nCD64; *P* = .8226; n = 8). On flow cytometry plots (Figure 8A2,B2), when Brefeldin A was added, there was no CD169 and CD64 extracellular expressions.

However, when Brefeldin A was added and the intracellular expression of both biomarkers was assessed (Figure 7A3,B3), significant intracellular increases were observed of mCD169 after IFN I stimulation (*P* = .0010; n = 8) and of nCD64 after IFN II stimulation (*P* = .0149; n = 8). Flow cytometry plots (Figure 8A3,B3) showed that both biomarkers were expressed.

These results confirmed that rapid kinetics of biomarker expressions were observed because of direct activation of the monocytes or neutrophils by the IFNs.

### 3.5 IFNs type I and 2 play specific and independent roles in CD169 and CD64 expression

**Figure 9:**
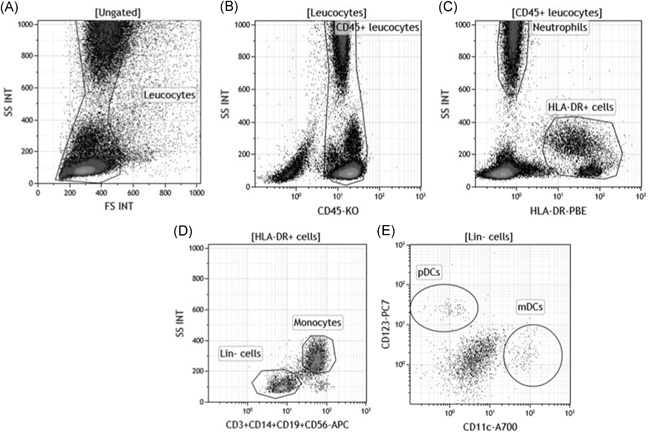
Monocytes, neutrophils, myeloid dendritic cells (mDCs) and plasmacytoid (pDCs) isolation by flow cytometry. A, First, leukocytes were isolated from red blood cells or debris or apoptotic cells based on their typical side (SS INT) and forward scatter (FS INT). B, Leukocytes CD45+ were gated out of them on their positive CD45 expression. C, Using the HLA‐DR expression on this population, negative neutrophils were separated from positive HLA‐DR cells, including B lymphocytes, monocytes, and DCs. D, Gated out from this last population, the lineage composed of CD3, CD14, CD19, and CD56 made the distinction between the positive monocytes and the lineage negative (lin‐) cells, including DCs. E, DCs were finally divided in both cellular subtypes: CD11c+CD123− mDCs or CD11c−CD123+ plasmacytoid DCs (pDCs)

The third part of the study was to decipher the cellular actors involved in the expression of both CD169 and CD64 biomarkers and which pathways they activate. Four cellular actors of the innate immune system were considered to have potential implications in these activations: monocytes, neutrophils, and both special myeloid (mDCs) and plasmacytoid (pDCs) subtypes of DCs (Figure 9).

**Figure 10:**
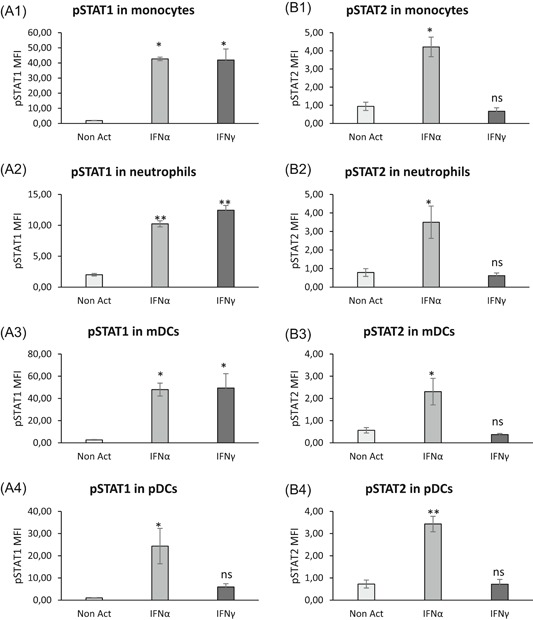
Cellular actors and activation pathways after interferon (IFN) stimulation. Whole blood of three donors was coincubated for 15 minutes at 37°C with either no IFN (Non Act; in white), or one type I IFN (IFNα1; in light gray) or one type II IFN (IFNγ; in the dark gray). Phosphoepitope staining of the activated blood was performed with the dendritic cells (DCs) antibody mixture. Results were expressed as averages of median of fluorescence intensities (MFI) of phosphorylated STAT1 (pSTAT1) (A1‐A4) and of pSTAT2 (B1‐B4) on monocytes (Figure 9A1,B1), neutrophils (Figure 9A2,B2), myeloid DCs (mDCs) (Figure 9A3,B3) and plasmacytoid DCs (pDCs) (Figure 9A4,B4). The comparison was made using a paired Tukey test, for which *P* value was considered either not statistically significant above .05 (NS) or statistically significant under .05 (*) or under .01 (**). NS, not significant

Intracellular expressions of both phosphorylated STAT1 and STAT2 were assessed in the four cellular types to study actors and pathways involved when whole blood was coincubated with IFNs (Figure 10). Detailed values are given in Table S5, and results are supported by flow cytometry plots presented in Figure 11.

Figure 11Flow cytometry expressions of phospho‐epitopes. The whole blood of one donor was coincubated for 15 minutes at 37°C with either no interferon (IFN) (Non Act), or one type I IFN (IFNα1) or one type II IFN (IFNγ). Phosphoepitope staining of the activated blood was performed with the dendritic cells (DCs) antibody mixture. Results were presented as histograms of phosphorylated STAT1 (pSTAT1) (Figure 10A) and of pSTAT2 (Figure 10B) on monocytes (Mo), neutrophils (Ne), myeloid DCs (mDCs), and plasmacytoid DCs (pDCs)

**Figure:**
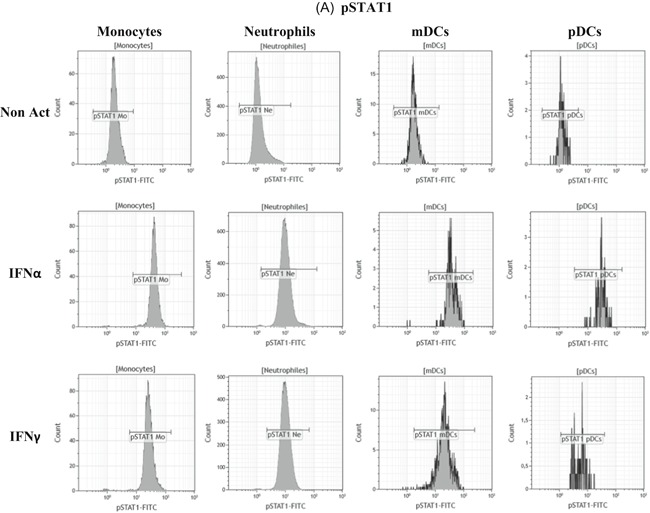

**Figure:**
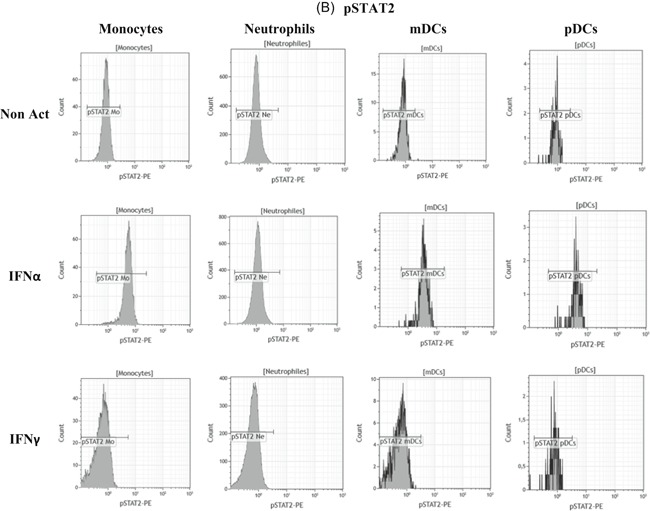

In comparison to nonactivated whole blood, pSTAT1 and pSTAT2 expressions were significantly increased in monocytes (pSTAT1: *P* = .0027; pSTAT2: *P* = .0032; n = 3), neutrophils (pSTAT1: *P* = .0002; pSTAT2: *P* = .0441; n = 3), mDCs (pSTAT1: *P* = .0337; pSTAT2: *P* = .0435; n = 3) and pDCs (pSTAT1: *P* = .0406; pSTAT2: *P* = .0016; n = 3) when blood was incubated with IFNα.

Conversely, only pSTAT1 expression was significantly increased in monocytes (*P* = .0030; n = 3), neutrophils (*P* < .0001; n = 3) and mDCs (*P* = .0297; n = 3) when blood was incubated with IFNγ.

These results confirm that IFNα triggered a double activation of pSTAT1 and pSTAT2 resulting in CD169 expression on monocytes, whereas IFNγ triggered a unique pSTAT1 activation resulting in CD64 expression on neutrophils.

**Figure 12:**
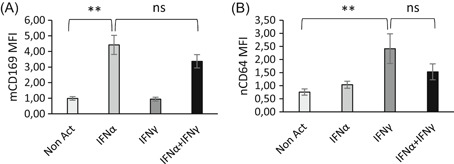
Combined effects of interferons (IFNs) on CD169 and CD64 expressions. Whole blood of six donors was coincubated for 18 hours at 37°C with either no IFN (Non Act; in white), or one type I IFN (IFNα1; in light gray) or one type II IFN (IFNγ; in the dark gray) or a combination of both IFNs (IFNα1 + IFNγ; in black). Extracellular staining of the activated blood was performed with the CD64‐CD169/infections antibody mixture. Results were expressed as averages ± standard deviations of the median of fluorescence intensities (MFI) of CD169 on monocytes (mCD169) (Figure 11A) and of CD64 on neutrophils (nCD64) (Figure 11B). The comparison was made using a paired Tukey test, for which *P* value was considered either not statistically significant above .05 (NS) or statistically significant under .05 (*) or under .01 (**). NS, not significant

Finally, the independence of both activation pathways was further deciphered by studying the effects of combined IFNs on biomarker expressions (Figure 12). Detailed values are given in Table S6.

When IFNα and IFNγ were concomitantly activating the blood, it seemed that IFNγ inhibited the IFNα‐induced mCD169 level because a slight (but nonsignificant) decrease was observed with the IFNα + IFNγ combination (3.4 ± 0.9; *P* = .1154; n = 6) in comparison to IFNα alone (4.4 ± 1.2).

Conversely, it seemed that IFNα inhibited the IFNγ‐induced nCD64 level because a slight (but nonsignificant) decrease was observed with the IFNα + IFNγ combination (1.5 ± 0.6; *P* =  .1355; n = 6) in comparison to IFNγ alone (2.4 ± 1.1).

These preliminary results indicated that the expression of both biomarkers relies on specific and independent activation pathways, depending on the IFN produced and the cell type.

## 4 DISCUSSION

The clinical distinction between viral and bacterial infections is an important issue for practitioners,^18^ as discrimination is often hard to establish based on clinical signs or currently measured biomarkers. Unfortunately, biomarkers are not currently combined into an application that is sensitive and specific enough for routine diagnosis. Therefore, when in doubt, antibiotic treatments are frequently prescribed, contributing to the dangerous rise of antibiotic resistance.^19^ The purpose of the study was to decipher pathways of two cell surface receptors described in infections, CD64 expressed on neutrophils and CD169 expressed on monocytes, and further establish their link with IFN, the main actor in antiviral or antibacterial responses.

CD64 plays a key role in bacterial phagocytosis, clearance of immune complexes, antigen presentation, and cytokine release.^20^ Increased neutrophil CD64 expression has been proposed as a biomarker of bacterial infection with superior performance^6^, ^21^, ^22^, ^23^, ^24^, ^25^ and even more strongly with sepsis diagnosis or acute infections.^26^, ^27^, ^28^ Conversely, CD169 is an endocytic receptor that recognizes sialylated molecules exposed on virus membrane gangliosides, binds to them to capture and internalize the virus, and either participates in its clearance or enhances its transmission and infectivity.^11^, ^29^ CD169 is mostly described as a resident macrophage marker and is not expressed in blood under healthy homeostasis. Only a few reports have pointed out its expression on monocytes upon diverse pathogenic processes, including rhinovirus infection, porcine reproductive, and respiratory syndrome virus infection and in certain inflammatory diseases such as multiple sclerosis, atherosclerosis, rheumatoid arthritis, and systemic lupus erythematosus, all being known to involve IFN I production, and in patients with viral infections such as Epstein‐Barr virus‐associated enteritis and human immunodeficiency virus (HIV) infection. Moreover, upregulated expression of CD169 in monocytes in HIV‐1‐infected patients is associated with high viral loads.^12^, ^29^, ^30^, ^31^

The rationale for the study was that immune system status is a reliable indicator of what happens at a cellular level for the recognition, elimination, and defense from what is foreign.^1^ On one hand, anatomical barriers, such as skin, mucous membranes, gastric acidity or any epithelium, are interesting because they represent the first defense against infectious agents. On the other hand, when a microorganism crosses one of these barriers, the cellular response induced by the innate immune system involves powerful actors, among them IFN, which is identified as acting primarily against pathogens. It was thus interesting to explore the functional link between IFN and both biomarkers.

In addition to IFN, several classes of activators were tested. However, only IFN was demonstrated to significantly induce direct mCD169 and nCD64 expressions after 7 hours of incubation. Particularly it was demonstrated that CD169 was increased on monocytes after IFN I stimulation, and CD64 was increased on neutrophils after IFN II stimulation. IFNs are described as three distinct classes (type I, II, and III).^32^ All act through the same mechanisms of action by the activation of IFN‐stimulated genes and microRNAs but are distinguished by their differing receptors, structural features, and biological activities according to the kind of infection that occurs in the human body.

IFN type I is principally described in viral infections to have immunomodulatory, antiproliferative, and antiviral functions after being secreted by almost all cell types.^4^ Some teams^33^ have further demonstrated that, among IFN I, some subtypes could be more or less effective, but no differences were observed in the present study. IFN type II is found in bacterial infections. Initially, only CD4+ T‐helper cells, CD8+ cytotoxic lymphocytes, and natural killer cells were believed to exclusively produce IFNγ, but there is now evidence that other cells, such as B cells, natural killer T cells, and professional antigen‐presenting cells also secrete IFNγ.^34^

The role of IFN III was not investigated in this study as it has not been correlated to biomarker expression. Type III IFN are a family of three proteins named IFNλ1, λ2, and λ3. These proteins were previously known as IL‐29, IL‐28A, and IL‐28B, respectively.^32^ The IFNλ3 gene is transcribed in the opposite direction of the IFNλ1 and IFNλ2 genes, but all act like IFN I by activating the Janus kinase (JAK)/STAT pathway. As it also stimulates, in turn, the phosphorylation of STAT1 and STAT2, a hypothesis needs to be investigated about the potential role of IFN III in mCD169 expression.

All other potential activators evaluated had no significant direct impact on either biomarker expression. However, the stimulation of whole blood by bacterial components such as LPS is normally known to induce CD64 expression on the surface of neutrophils.^6^, ^8^ Also, certain toll‐like receptor ligands, such as Poly IC, are described to induce CD169 expression on the surface of monocytes.^35^ And finally, ILs are sometimes related to IFN production.^3^ Later time points did show some of these expected effects, but this could be explained by multiple indirect pathways, and deserves further investigation.

Precise kinetics were established. Results obtained strongly correlated with the literature. Indeed, it is demonstrated that nCD64 upregulation is elevated to three times the normal level after IFNγ stimulation and occurs within a short time scale of 6 hours for cell surface expression.^8^ Also, detectable messenger RNA (mRNA) increases by northern blot analysis in 1 to 3 hours.^24^ In parallel, CD169 protein expression, and also mRNA, is demonstrated to be significantly increased after viral infection in vivo.^30^ To our knowledge, this is the first time its expression was demonstrated to be directly induced by IFN. This was expected, as monocytes and neutrophils are known to have IFN receptors on their surfaces that in turn activate the JAK/STAT pathways. IFN I and IFN II seem to have cross‐inhibitory effects on each other regarding the induction of CD169 or CD64, respectively, reflecting the immune system orientation in response to a virus or bacteria.

Finally, the assessment of the biomarkers was made according to a newly described procedure of flow cytometry.^14^ Indeed, one of the prerequisites was that the biomarkers chosen could be easily quantified when further applied in a clinical context. That was a major issue with IFN, as titration relies on complex techniques with often biased or difficult measurements.^4^ As immediate downstream events, CD64 and CD169 expression are supposed to parallel the levels of IFNs, with the advantage of being easily measured in whole blood, with a one‐step sample preparation procedure, and levels assessed in 15 minutes, all advantages for practitioners. It could even be further applied at the patient's bedside.

Of course, the study has some limitations. First, activators have all been titrated in preparation of the study to maximize their effects, and have doses close to what happens physiologically. Furthermore, several whole blood volumes have been tested to best mimic what happens in vivo when activators are put in contact with host cells. Despite this, a cellular environment is hardly reproducible because of the large spectrum of interactions that could exist or the diversity of factors impacting on pathways, even more in the case of such complex pathologies like infections. Moreover, pathogens involved in vivo could sometimes deflect the cellular machinery leading to a different or inexistent host response with IFNs. Finally, CD169 and CD64 levels have only been quantified by flow cytometry, whereas it would have been interesting to measure it with other techniques, knowing for instance that the biomarkers mRNA levels also correlate with IFN activation. These results should be investigated further.

## 5 CONCLUSION

The goal of the study was to try to understand the kinetics of CD169 expression on monocytes and CD64 expression on neutrophils during the course of a viral and bacterial infection, as they have not been well studied to date. The functional link between markers and IFN were explored to help identify the potential relevance of using them in a clinical context. In response to a bacterial infection, IFNγ directly induces, in 6 hours, nCD64 expression via pSTAT1, whereas in the case of a viral infection, IFNα directly induces, in 8 hours, mCD169 expression via pSTAT1 and pSTAT2. Both biomarkers were further demonstrated to be independently activated by IFN, preventing false results from interactions, and to be rapidly expressed, due to direct and easily detectable pathways established in response by the host in the first hours of the infection.

## CONFLICT OF INTERESTS

Pénélope Bourgoin, Géraldine Biéchelé, Inès Ait Belkacem, and Fabrice Malergue are Beckman Coulter employees. Beckman Coulter and the Beckman Coulter product and service marks mentioned herein are trademarks or registered trademarks of Beckman Coulter, Inc, in the United States and other countries. All other trademarks are the property of their respective owners.

## Supporting information

Supporting information

## Data Availability

The authors certify that this manuscript reports original clinical research data. Individual data that underlie the results reported in this article are available from the corresponding author following publication, including the study report and study protocol. Additional data are available upon reasonable request.
