# Supplementary material for: Role of the interferons in CD64 and CD169 expressions in whole blood: Relevance in the balance between viral‐ or bacterial‐oriented immune responses
Source: Immun Inflamm Dis. 2020 Feb 7;8(1):106–23. doi: 10.1002/iid3.289 (PMC7016842; doi:10.1002/iid3.289)
Supplement: Supplementary file 1 — Supporting information [file IID3-8-106-s001.pdf]

SUPPORTING INFORMATION.

Supplementary Table 1. Detailed values of CD169 and CD64 expressions after blood stimulation. *Whole blood of 3 donors was co-incubated for 3 (A1 and B1), 5 (A2 and B2), 7 (A3 and B3) or 24 (A4 and B4) hours at 37°C with either no activator (Non Act), or interleukins (IL-2, IL-6, IL-12, IL-18, IL-12+IL-18) or infectious extracts (Poly IC, LPS) or interferons (IFNα1, IFNγ). Extracellular staining of the activated blood was performed with the CD64-CD169/Infections antibody mixture. Results are expressed as means ± standard deviations (stdev) of Median of Fluorescence Intensities (MFI) of CD169 on monocytes (mCD169) (supplementary tables 1A) and of CD64 on neutrophils (nCD64) (supplementary tables 1B). Comparison was made using a Dunnett's control test, with the Non Act condition used as control, and p-value was considered statistically significant under 0.05 (in bold with *).*

(A1) mCD169 MFI - 3h

Activator	Non Act	IL-2	IL-6	IL-12	IL-18	IL-12 + IL-18	Poly IC	LPS	IFNα	IFNγ
Mean (± stdev)	0,89 (± 0,05)	0,92 (± 0,06)	0,86 (± 0,03)	0,87 (± 0,01)	0,90 (± 0,12)	0,83 (± 0,03)	0,83 (± 0,07)	1,15 (± 0,05)	0,92 (± 0,02)	0,89 (± 0,07)
Dunett p-value	/	0,9985	0,9855	0,9978	1	0,7791	0,6947	**0,0004***	0,999	1

(A2) mCD169 MFI - 5h

Activator	Non Act	IL-2	IL-6	IL-12	IL-18	IL-12 + IL-18	Poly IC	LPS	IFNα	IFNγ
Mean (± stdev)	0,89 (± 0,05)	0,94 (± 0,01)	0,81 (± 0,13)	0,71 (± 0,28)	0,88 (± 0,09)	0,88 (± 0,1)	0,90 (± 0,09)	1,16 (± 0,17)	1,15 (± 0,24)	0,90 (± 0,13)
Dunett p-value	/	0,9998	0,9771	0,6049	1	1	1	0,1467	0,1872	1

(A3) mCD169 MFI - 7h

Activator	Non Act	IL-2	IL-6	IL-12	IL-18	IL-12 + IL-18	Poly IC	LPS	IFNα	IFNγ
Mean (± stdev)	0,89 (± 0,05)	1,30 (± 0,09)	1,07 (± 0,18)	0,97 (± 0,13)	1,04 (± 0,09)	1,00 (± 0,1)	1,25 (± 0,1)	1,39 (± 0,26)	1,81 (± 0,57)	1,12 (± 0,11)
Dunett p-value	/	0,2011	0,9219	0,9999	0,9674	0,9964	0,3324	0,0857	**0,0006***	0,7634

(A4) mCD169 MFI - 24h

Activator	Non Act	IL-2	IL-6	IL-12	IL-18	IL-12 + IL-18	Poly IC	LPS	IFNα	IFNγ
**Mean (± stdev)**	0,89 (± 0,05)	0,90 (± 0,1)	1,08 (± 0,22)	1,05 (± 0,09)	0,92 (± 0,08)	0,99 (± 0,24)	1,43 (± 0,38)	0,76 (± 0,16)	2,37 (± 1,44)	0,79 (± 0,15)
**Dunett p-value**	/	1	0,9991	1	1	1	0,6989	1	**0,0103***	1

(B1) nCD64 MFI - 3h

Activator	Non Act	IL-2	IL-6	IL-12	IL-18	IL-12 + IL-18	Poly IC	LPS	IFNα	IFNγ
**Mean (± stdev)**	0,96 (± 0,09)	0,96 (± 0,12)	0,96 (± 0,09)	0,97 (± 0,13)	0,96 (± 0,13)	0,96 (± 0,12)	0,95 (± 0,13)	1,29 (± 0,14)	0,99 (± 0,13)	0,98 (± 0,17)
**Dunett p-value**	/	1	1	1	1	1	1	**0,0264***	0,9999	1

(B2) nCD64 MFI - 5h

Activator	Non Act	IL-2	IL-6	IL-12	IL-18	IL-12 + IL-18	Poly IC	LPS	IFNα	IFNγ
**Mean (± stdev)**	0,96 (± 0,09)	0,98 (± 0,12)	0,92 (± 0,11)	0,87 (± 0,1)	0,98 (± 0,15)	1,01 (± 0,15)	0,96 (± 0,16)	1,29 (± 0,22)	1,10 (± 0,2)	1,33 (± 0,18)
**Dunett p-value**	/	1	0,9999	0,9942	1	0,9997	1	0,0841	0,8208	**0,0437***

(B3) nCD64 MFI - 7h

Activator	Non Act	IL-2	IL-6	IL-12	IL-18	IL-12 + IL-18	Poly IC	LPS	IFNα	IFNγ
**Mean (± stdev)**	0,96 (± 0,09)	1,08 (± 0,14)	0,99 (± 0,04)	0,98 (± 0,04)	1,05 (± 0,1)	1,31 (± 0,16)	1,04 (± 0)	1,34 (± 0,04)	1,29 (± 0,2)	1,86 (± 0,41)
**Dunett p-value**	/	0,9515	1	1	0,991	0,1228	0,9962	0,0725	0,1597	**< 0,0001***

(B4) nCD64 MFI - 24h

Activator	Non Act	IL-2	IL-6	IL-12	IL-18	IL-12 + IL-18	Poly IC	LPS	IFNα	IFNγ
**Mean (± stdev)**	0,96 (± 0,09)	1,61 (± 0,2)	0,97 (± 0,18)	1,17 (± 0,02)	1,33 (± 0,55)	2,17 (± 1,9)	1,06 (± 0,24)	1,39 (± 0,47)	1,28 (± 0,55)	2,40 (± 1,73)
**Dunett p-value**	/	0,9364	1	1	0,998	0,4562	1	0,9941	0,9993	0,2798

SUPPORTING INFORMATION.

Healthy fresh blood

(A) Large gating

(B) Restricted gating

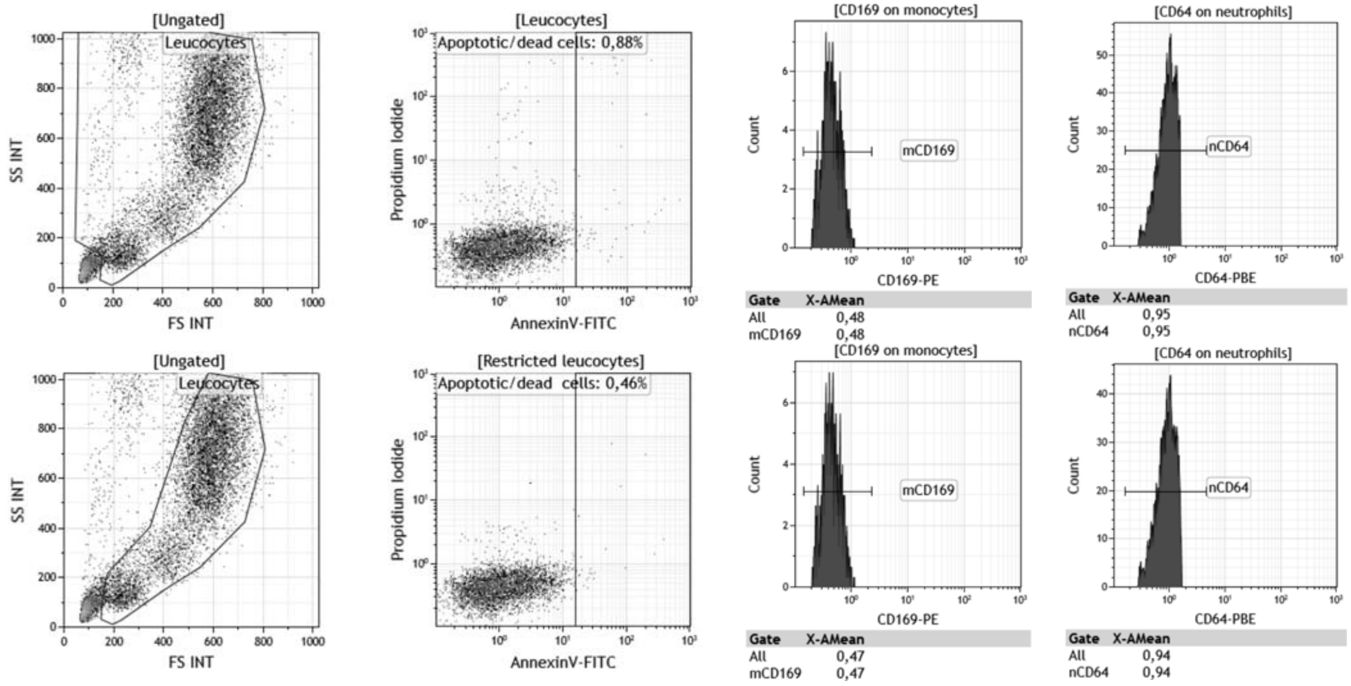

Healthy incubated blood

(A) Large gating

(B) Restricted gating

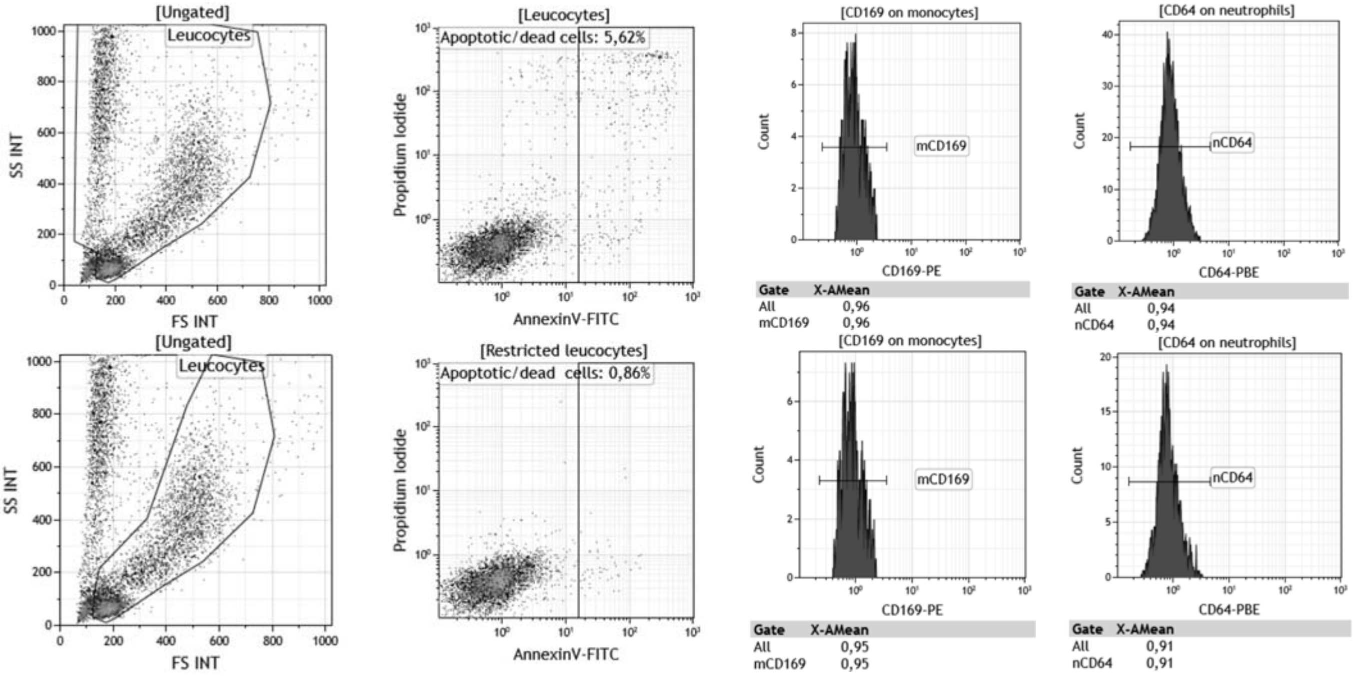

IFNα-incubated blood

(A) Large gating

(B) Restricted gating

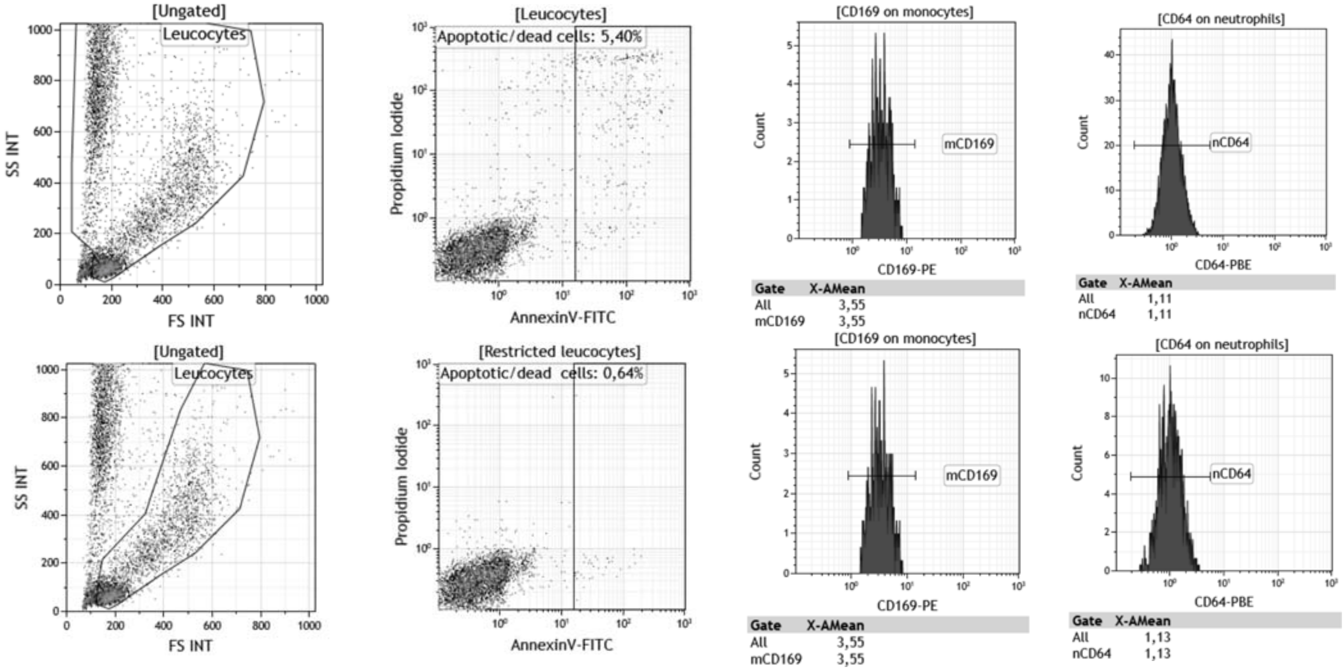

IFNγ-incubated blood

(A) Large gating

(B) Restricted gating

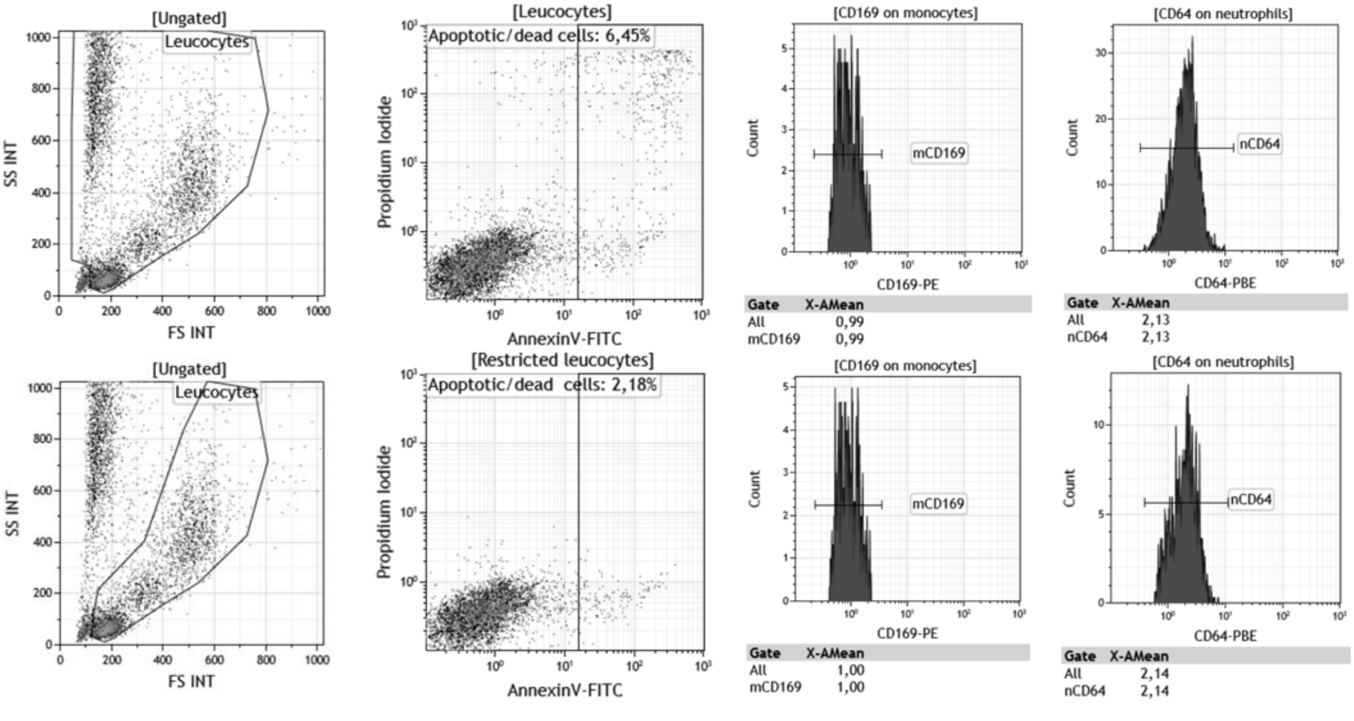

Supplementary Figure 1. Apoptosis impact on CD169 and CD64. *Whole blood of 1 donor was tested fresh (healthy fresh blood) or co-incubated for 24 hours at 37°C with either no activator (healthy incubated blood) or type I interferon (IFNα-incubated blood) or type II interferon (IFNγ-incubated blood). Extracellular staining of all samples was performed either with Annexin V-FITC plus Propidium Iodide (PI) or with the CD64-CD169/Infections antibody mixture. Leucocytes were selected on their typical forward (FS) and side (SS) scatter, largely including all cells (large gating; supplementary figures 1A) or narrowly selecting only cells with high FS (restricted gating; supplementary figures 1B).*

Results show that, when tested fresh, whole blood from a healthy donor has less than 1% of apoptotic (Annexin V+ PI-) or dead (Annexin V+ PI+) cells, whatever the leucocyte gating, with expressions of mCD169 and nCD64 about 0.5 and 0.9, respectively.

When this same sample is incubated 24 hours at 37°C with no activator or IFN I or IFN II, about 5-6% of apoptotic or dead cells appear when considering all cells. However, when using a restricted gating strategy, only 1-2% of apoptotic or dead cells remain for all incubated samples.

Results indicate that using a restricted gating strategy prevents including most apoptotic or dead cells in the analysis. Further, taking this precaution has no impact on the two biomarker levels: mCD169 is about 1.0, 3.6 and 1.0 in a non-activated, an IFN I-activated and an IFN II-activated blood, respectively, whatever the gating strategy, while nCD64 is about 0.9, 1.1 and 2.1 in a non-activated, an IFN I-activated and an IFN II-activated blood, respectively, whatever the gating strategy.

SUPPORTING INFORMATION.

Supplementary Table 2. Detailed values of CD169 and CD64 expressions after interferon stimulation. *Whole blood of 4 donors was co-incubated for 15 hours at 37°C with either no interferon (Non Act), or type I interferons (IFNα1, IFNα2, IFNα3, IFNα4, IFNα5, IFNα6, IFNα7, IFNα8, IFNα9, IFNα10, IFNα11, IFNα12, IFNβ, IFNω) or type II interferon (IFNγ). Extracellular staining of the activated blood was performed with the CD64-CD169/Infections antibody mixture. Results are expressed as means ± standard deviations (stdev) of Median of Fluorescence Intensities (MFI) of CD169 on monocytes (mCD169) (supplementary table 2A) and of CD64 on neutrophils (nCD64) (supplementary table 2B). Comparison was made using a Dunnett's control test, with the Non Act condition used as control, and p-value was considered statistically significant under 0.05 (in bold with *).*

(A) mCD169 MFI

Activator	Non Act	IFNα1	IFNα2	IFNα3	IFNα4	IFNα5	IFNα6	IFNα7
**Mean (± stdev)**	1,7 (± 0,5)	8,7 (± 1,9)	7,7 (± 0,9)	8,3 (± 1,3)	8,4 (± 1,1)	7,8 (± 1)	8,2 (± 1,1)	10,2 (± 1)
**Dunnett p-value**	/	**< 0,0001***	**< 0,0001***	**< 0,0001***	**< 0,0001***	**< 0,0001***	**< 0,0001***	**< 0,0001***

(A) mCD169 MFI

Activator	IFNα8	IFNα9	IFNα10	IFNα11	IFNα12	IFNβ	IFNω	IFNγ
**Mean (± stdev)**	9,6 (± 0,9)	9,4 (± 1,4)	9,7 (± 1,3)	6,6 (± 0,4)	7,8 (± 0,9)	7,8 (± 0,6)	9,6 (± 1,9)	3,3 (± 1,5)
**Dunnett p-value**	**< 0,0001***	**< 0,0001***	**< 0,0001***	**0,0005***	**0,0042***	**< 0,0001***	**< 0,0001***	0,1979

(B) nCD64 MFI

Activator	Non Act	IFNα1	IFNα2	IFNα3	IFNα4	IFNα5	IFNα6	IFNα7
**Mean (± stdev)**	1,1 (± 0,3)	1,7 (± 0,5)	1,6 (± 0,5)	1,6 (± 0,5)	1,6 (± 0,5)	1,6 (± 0,5)	1,6 (± 0,6)	1,7 (± 0,5)
**Dunnett p-value**	/	0,8185	0,8897	0,8743	0,9318	0,9199	0,9384	0,7835

(B) nCD64 MFI

Activator	IFNα8	IFNα9	IFNα10	IFNα11	IFNα12	IFNβ	IFNω	IFNγ
**Mean (± stdev)**	1,7 (± 0,6)	1,7 (± 0,6)	1,8 (± 0,6)	1,5 (± 0,6)	1,7 (± 0,7)	1,7 (± 0,5)	1,7 (± 0,6)	2,9 (± 0,8)
**Dunnett p-value**	0,6995	0,7549	0,5375	0,9882	1,0000	0,8147	0,6821	**0,0008***

SUPPORTING INFORMATION.

(A)

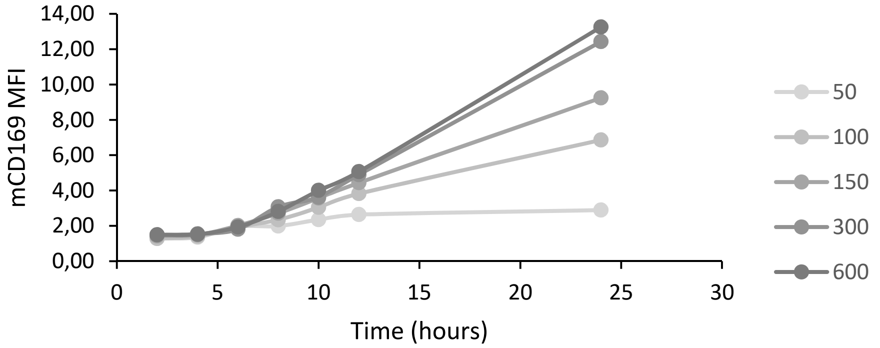

(B)

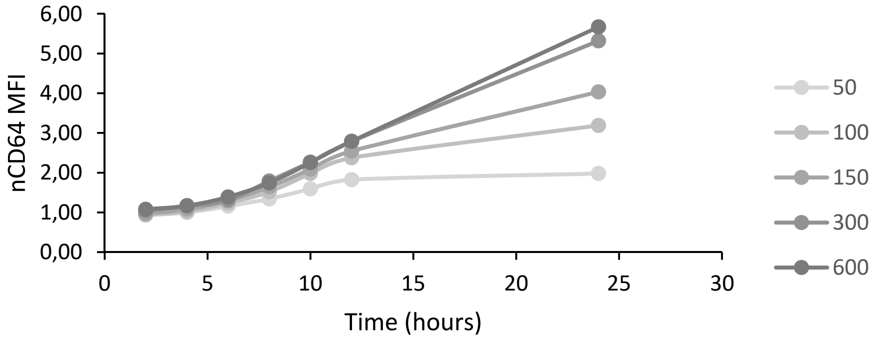

Supplementary Figure 2. CD169 and CD64 expressions in different interferon-activated blood volumes. *Whole blood varying from 50 µL to 600 µL (light to dark grey) of 2 donors was co-incubated for 2 to 24 hours at 37°C with either one type I interferon (IFNα1) or one type II interferon (IFNγ). Extracellular staining of all activated blood volumes was performed with the CD64-CD169/Infections antibody mixture. Results were expressed as averages of Median of Fluorescence Intensities (MFI) of CD169 on monocytes (mCD169) when blood volumes were activated with IFNα1 (figure 3A) and of CD64 on neutrophils (nCD64) when blood volumes were activated with IFNγ (figure 3B). Comparison was made using an ANOVA test, for which p-value is considered statistically significant under 0.05. When p-value was under 0.05, comparison has been made using a paired Tukey test, for which p-value is also considered statistically significant under 0.05.*

Results of the ANOVA test show that up to 8 hours of incubation with IFNα1, blood volume has no significant impact on mCD169 level (2h – p: 0.4733; 4h – p: 0.6690; 6h – p: 0.4443; 8h – p: 0.0502). However, after 10 hours of incubation, blood volumes give significantly different mCD169 levels (10h – p: 0.0004; 12h – p: 0.0024; 24h – p: 0.0004), meaning that long incubations with interferon implies to be careful with blood volume used for activation.

Results of the paired Tukey test (p-values not detailed) show that after 10 or 12 hours of incubation, 150, 300 and 600 µL are blood volumes significantly giving higher mCD169 levels than 50 or 100 µL, but equivalent between them. After 24 hours, 300 and 600 µL of blood have significant impact on mCD169 level. All these results converge to demonstrate that a minimal initial blood volume of 300 µL is required when mCD169 is assessed in whole blood activated by IFNα1.

In the same way, results of the ANOVA test show that up to 6 hours of incubation with IFNγ, blood volume has no significant impact on nCD64 level (2h – p: 0.2100; 4h – p: 0.2036; 6h – p: 0.1493). However, after 8 hours of incubation, blood volumes give significantly different nCD64 levels (8h – p: 0.0046; 10h – p: 0.0088; 12h – p: 0.0047; 24h – p: 0.0027), with results of the paired Tukey test (not detailed) showing that 150 µL of initial blood volume is the minimum required for assessing nCD64 in whole blood activated by IFNγ.

SUPPORTING INFORMATION.

Supplementary Table 3. Detailed values of CD169 and CD64 kinetics after interferon stimulation. *Whole blood of 4 donors was co-incubated for 2 to 12 hours at 37°C with either no interferon (Non Act), or one type II interferon (IFNγ) or one type I interferon (IFNα1). Extracellular staining of the activated blood was performed with the CD64-CD169/Infections antibody mixture. Results were expressed as means ± standard deviations (stdev) of Median of Fluorescence Intensities (MFI) of CD169 on monocytes (mCD169) (supplementary table 3A1) and of CD64 on neutrophils (nCD64) (supplementary table 3B1). Comparison was made using an ANOVA test, for which p-value was considered statistically significant under 0.05 (in bold with *). When p-value was under 0.05, detailed comparison between each hours was made for mCD169 (supplementary table A2) and nCD64 (supplementary table B2), using a paired Tukey test, for which p-value was also considered statistically significant under 0.05 (in bold with *).*

(A1) mCD169 MFI

Time (h)	2	4	6	8	10	12	ANOVA p-value
**Non Act Mean (± stdev)**	1,45 (± 0,13)	1,40 (± 0,17)	1,37 (± 0,14)	1,31 (± 0,10)	1,40 (± 0,05)	1,58 (± 0,15)	0,1299
**IFNγ Mean (± stdev)**	1,44 (± 0,19)	1,31 (± 0,13)	1,33 (± 0,16)	1,33 (± 0,14)	1,48 (± 0,15)	1,58 (± 0,18)	0,1706
**IFNα Mean (± stdev)**	1,49 (± 0,14)	1,53 (± 0,18)	1,87 (± 0,12)	2,94 (± 0,35)	3,81 (± 0,25)	4,98 (± 0,18)	**< 0,0001***

(A2) mCD169 MFI

IFNα Tukey p-value	2	4	6	8	10	12
**2**	/	0,9997	0,1806	**< 0,0001***	**< 0,0001***	**< 0,0001***
**4**	0,9997	/	0,2815	**< 0,0001***	**< 0,0001***	**< 0,0001***
**6**	0,1806	0,2815	/	**< 0,0001***	**< 0,0001***	**< 0,0001***
**8**	**< 0,0001***	**< 0,0001***	**< 0,0001***	/	**0,0003***	**< 0,0001***
**10**	**< 0,0001***	**< 0,0001***	**< 0,0001***	**0,0003***	/	**< 0,0001***
**12**	**< 0,0001***	**< 0,0001***	**< 0,0001***	**< 0,0001***	**< 0,0001***	/

(B1) nCD64 MFI

Time (h)	2	4	6	8	10	12	ANOVA p-value
**Non Act Mean (± stdev)**	1,05 (± 0,06)	1,11 (± 0,04)	1,10 (± 0,08)	1,09 (± 0,06)	1,13 (± 0,11)	1,20 (± 0,16)	0,3776
**IFNγ Mean (± stdev)**	1,06 (± 0,02)	1,16 (± 0,04)	1,35 (± 0,10)	1,77 (± 0,03)	2,26 (± 0,04)	2,79 (± 0,05)	**< 0,0001***
**IFNα Mean (± stdev)**	1,09 (± 0,02)	1,10 (± 0,03)	1,11 (± 0,09)	1,19 (± 0,10)	1,14 (± 0,02)	1,22 (± 0,02)	0,2456

(B2) nCD64 MFI

IFNγ Tukey p-value	2	4	6	8	10	12
**2**	/	0,1212	**< 0,0001***	**< 0,0001***	**< 0,0001***	**< 0,0001***
**4**	0,1212	/	**0,0011***	**< 0,0001***	**< 0,0001***	**< 0,0001***
**6**	**< 0,0001***	**0,0011***	/	**< 0,0001***	**< 0,0001***	**< 0,0001***
**8**	**< 0,0001***	**< 0,0001***	**< 0,0001***	/	**< 0,0001***	**< 0,0001***
**10**	**< 0,0001***	**< 0,0001***	**< 0,0001***	**< 0,0001***	/	**< 0,0001***
**12**	**< 0,0001***	**< 0,0001***	**< 0,0001***	**< 0,0001***	**< 0,0001***	/

SUPPORTING INFORMATION.

Supplementary Table 4. Detailed values of direct or indirect CD169 and CD64 kinetics after interferon stimulation. *Whole blood of 8 donors was co-incubated for 2 to 24 hours at 37°C with either no interferon (Non Act), or one type II interferon (IFNγ) or one type I interferon (IFNα1). On one hand, biomarker extracellular expressions (Extra.) were assessed in activated whole blood without Brefeldin A (Bref. A), a Golgi apparatus blocker, (supplementary table A1 and B1) or with Brefeldin A (supplementary table A2 and B2). On the other hand, intracellular expressions (Intra.) were evaluated with Brefeldin A only (supplementary table A3 and B3). All stainings were performed with the CD64-CD169/Infections antibody mixture. Results were expressed as means ± standard deviations (stdev) of Median of Fluorescence Intensities (MFI) of CD169 on monocytes (mCD169) (supplementary tables 4A) and of CD64 on neutrophils (nCD64) (supplementary tables 4B). Comparison was made using an ANOVA test, for which p-value is considered statistically significant under 0.05 (in bold with *).*

(A1) mCD169 MFI (Extra. Without Bref. A)

Time (h)	2	4	6	8	ANOVA p-value
**Non Act Mean (± stdev)**	0,83 (± 0,01)	0,79 (± 0,17)	0,79 (± 0,18)	0,87 (± 0,10)	0,1236
**IFNγ Mean (± stdev)**	0,87 (± 0,35)	0,94 (± 0,39)	0,76 (± 0,67)	1,13 (± 0,71)	0,3641
**IFNα Mean (± stdev)**	0,88 (± 0,36)	0,96 (± 0,40)	1,05 (± 0,56)	1,90 (± 0,47)	**< 0,0001***

(A2) mCD169 MFI (Extra. With Bref. A)

Time (h)	2	4	6	8	ANOVA p-value
**Non Act Mean (± stdev)**	0,87 (± 0,01)	0,66 (± 0,17)	0,72 (± 0,14)	0,73 (± 0,22)	0,7002
**IFNγ Mean (± stdev)**	0,86 (± 0,04)	0,64 (± 0,13)	0,68 (± 0,15)	0,69 (± 0,11)	0,5019
**IFNα Mean (± stdev)**	0,83 (± 0,02)	0,88 (± 0,29)	0,71 (± 0,18)	1,01 (± 0,55)	0,3478

(A3) mCD169 MFI (Intra. With Bref. A)

Time (h)	2	4	6	8	ANOVA p-value
**Non Act Mean (± stdev)**	0,73 (± 0,04)	0,54 (± 0,07)	0,58 (± 0,12)	0,55 (± 0,20)	0,4334
**IFNγ Mean (± stdev)**	0,85 (± 0,01)	0,63 (± 0,13)	0,61 (± 0,11)	0,72 (± 0,23)	0,2969
**IFNα Mean (± stdev)**	0,79 (± 0,16)	0,98 (± 0,38)	1,64 (± 0,51)	2,48 (± 0,90)	**0,0010***

(B1) nCD64 (Extra. Without Bref. A)

Time (h)	2	4	6	8	ANOVA p-value
**Non Act Mean (± stdev)**	0,88 (± 0,35)	0,69 (± 0,38)	1,04 (± 0,54)	0,81 (± 0,47)	0,4284
**IFNγ Mean (± stdev)**	0,92 (± 0,35)	0,84 (± 0,39)	1,33 (± 0,67)	1,45 (± 0,71)	**0,0002***
**IFNα Mean (± stdev)**	0,87 (± 0,36)	0,77 (± 0,40)	1,10 (± 0,56)	0,99 (± 0,47)	0,0766

(B2) nCD64 (Extra. With Bref. A)

Time (h)	2	4	6	8	ANOVA p-value
**Non Act Mean (± stdev)**	0,85 (± 0,37)	0,96 (± 0,56)	0,96 (± 0,52)	0,95 (± 0,49)	0,9222
**IFNγ Mean (± stdev)**	0,84 (± 0,38)	0,96 (± 0,57)	0,97 (± 0,53)	1,00 (± 0,46)	0,8226
**IFNα Mean (± stdev)**	0,87 (± 0,37)	0,68 (± 0,33)	0,95 (± 0,48)	0,75 (± 0,36)	0,7568

(B3) nCD64 (Intra. With Bref. A)

Time (h)	2	4	6	8	ANOVA p-value
**Non Act Mean (± stdev)**	0,66 (± 0,26)	0,54 (± 0,27)	0,65 (± 0,27)	0,51 (± 0,27)	0,7130
**IFNγ Mean (± stdev)**	0,61 (± 0,20)	0,53 (± 0,13)	0,90 (± 0,35)	1,11 (± 0,34)	**0,0149***
**IFNα Mean (± stdev)**	0,65 (± 0,22)	0,54 (± 0,27)	0,66 (± 0,23)	0,61 (± 0,24)	0,8487

SUPPORTING INFORMATION.

Supplementary Table 5. Detailed values of cellular actors and activation pathways after interferon stimulation. *Whole blood of 3 donors was co-incubated for 15 minutes at 37°C with either no interferon (Non Act), or one type I interferon (IFNα1) or one type II interferon (IFNγ). Phospho-epitope staining of the activated blood was performed with the DCs antibody mixture. Results were expressed as means ± standard deviations (stdev) of Median of Fluorescence Intensities (MFI) of phosphorylated STAT1 (pSTAT1) (supplementary tables 5A1 to 5A4) and of pSTAT2 (supplementary tables 5B1 to 5B4) on monocytes (supplementary tables 5A1 and 5B1), neutrophils (supplementary tables 5A2 and 5B2), myeloid dendritic cells (mDCs) (supplementary tables 5A3 and 5B3) and plasmacytoid dendritic cells (pDCs) (supplementary tables 5A4 and 5B4). Comparison was made using a paired Tukey test, for which p-value was considered statistically significant under 0.05 (in bold with *).*

(A1) pSTAT1 MFI on monocytes

Activator	Non Act	IFNα	IFNγ
**Mean (± stdev)**	1,88 (± 0,16)	42,66 (± 1,16)	41,92 (± 7,34)
**Tukey p-value Activator vs Non Act**	/	**0,0027***	**0,0030***

(B1) pSTAT2 MFI on monocytes

Activator	Non Act	IFNα	IFNγ
**Mean (± stdev)**	0,94 (± 0,23)	4,22 (± 0,54)	0,67 (± 0,19)
**Tukey p-value Activator vs Non Act**	/	**0,0032***	0,8894

(A2) pSTAT1 MFI on neutrophils

Activator	Non Act	IFNα	IFNγ
**Mean (± stdev)**	2,00 (± 0,20)	10,24 (± 0,49)	12,45 (± 0,76)
**Tukey p-value Activator vs Non Act**	/	**0,0002***	**< 0,0001***

(B2) pSTAT2 MFI on neutrophils

Activator	Non Act	IFNα	IFNγ
**Mean (± stdev)**	0,79 (± 0,21)	3,50 (± 0,87)	0,62 (± 0,15)
**Tukey p-value Activator vs Non Act**	/	**0,0441***	0,9786

(A3) pSTAT1 MFI on mDCs

Activator	Non Act	IFNα	IFNγ
**Mean (± stdev)**	2,66 (± 0,42)	48,01 (± 5,80)	49,40 (± 12,92)
**Tukey p-value Activator vs Non Act**	/	**0,0337***	**0,0297***

(B3) pSTAT2 MFI on mDCs

Activator	Non Act	IFNα	IFNγ
**Mean (± stdev)**	0,57 (± 0,12)	2,31 (± 0,60)	0,37 (± 0,06)
**Tukey p-value Activator vs Non Act**	/	**0,0435***	0,9387

(A4) pSTAT1 MFI on pDCs

Activator	Non Act	IFNα	IFNγ
**Mean (± stdev)**	1,00 (± 0,15)	24,36 (± 7,97)	5,93 (± 1,48)
**Tukey p-value Activator vs Non Act**	/	**0,0406***	0,8013

(B4) pSTAT2 MFI on pDCs

Activator	Non Act	IFNα	IFNγ
**Mean (± stdev)**	0,73 (± 0,18)	3,43 (± 0,35)	0,72 (± 0,21)
**Tukey p-value Activator vs Non Act**	/	**0,0016***	0,9999

SUPPORTING INFORMATION.

Supplementary Table 6. Detailed values of combined effects of interferons on CD169 and CD64 expressions. *Whole blood of 6 donors was co-incubated for 18 hours at 37°C with either no interferon (Non Act), or one type I interferon (IFNα1) or one type II interferon (IFNγ) or a combination of both interferons (IFNα1+IFNγ). Extracellular staining of the activated blood was performed with the CD64-CD169/Infections antibody mixture. Results were expressed as means ± standard deviations (stdev) of Median of Fluorescence Intensities (MFI) of CD169 on monocytes (mCD169) (supplementary table 6A) and of CD64 on neutrophils (nCD64) (supplementary table 6B). Comparison was made using a paired Tukey test, for which p-value was considered statistically significant under 0.05 (in bold with *).*

(A) mCD169 MFI

**Activator**	**Non Act**	**IFNα**	**IFNγ**	**IFNα+IFNγ**
**Mean (± stdev)**	0,99 (± 0,23)	4,42 (± 1,23)	0,94 (± 0,25)	3,37 (± 0,86)
**Tukey p-value Activator vs Non Act**	/	**< 0,0001***	0,9996	**0,0002***
**Tukey p-value Activator vs IFNα**	**< 0,0001***	/	**< 0,0001***	0,1154

(B) nCD64 MFI

**Activator**	**Non Act**	**IFNα**	**IFNγ**	**IFNα+IFNγ**
**Mean (± stdev)**	0,76 (± 0,24)	1,04 (± 0,28)	2,41 (± 1,13)	1,53 (± 0,61)
**Tukey p-value Activator vs Non Act**	/	0,8858	**0,0019***	0,2184
**Tukey p-value Activator vs IFNγ**	**0,0019***	**0,0096***	/	0,1355
